# Enhanced Collateral Growth by Double Transplantation of Gene-Nucleofected Fibroblasts in Ischemic Hindlimb of Rats

**DOI:** 10.1371/journal.pone.0019192

**Published:** 2011-04-25

**Authors:** Ziyang Zhang, Alex Slobodianski, Wulf D. Ito, Astrid Arnold, Jessica Nehlsen, Shaoxiang Weng, Natalie Lund, Jihong Liu, José-Tomás Egaña, Jörn A. Lohmeyer, Daniel F. Müller, Hans-Günther Machens

**Affiliations:** 1 Department of Plastic Surgery and Hand Surgery, Faculty of Medicine, University Hospital Rechts der Isar, Technische Universität München, Munich, Germany; 2 Department of Plastic Surgery and Hand Surgery, University of Lübeck, Lübeck, Germany; 3 Cardiovascular Center Oberallgaeu, Academic Teaching Hospital, University of Ulm, Immenstadt, Germany; 4 Department of Cardiovascular Diseases, School of Medicine, Sir Run Run Shaw Hospital, Zhejiang University, Hangzhou, China; 5 Experimental Angiology, Medical Department II, University Hospital Lübeck, Lübeck, Germany; 6 Department of Urology, Tongji Hospital, Huazhong University of Science and Technology, Wuhan, China; 7 Facultad de Ciencias, Center for Genome Regulation, Universidad de Chile, Santiago, Chile; Istituto Dermopatico dell'Immacolata, Italy

## Abstract

**Background:**

Induction of neovascularization by releasing therapeutic growth factors is a promising application of cell-based gene therapy to treat ischemia-related problems. In the present study, we have developed a new strategy based on nucleofection with alternative solution and cuvette to promote collateral growth and re-establishment of circulation in ischemic limbs using double transplantation of gene nucleofected primary cultures of fibroblasts, which were isolated from rat receiving such therapy.

**Methods and Results:**

Rat dermal fibroblasts were nucleofected *ex vivo* to release bFGF or VEGF165 in a hindlimb ischemia model *in vivo*. After femoral artery ligation, gene-modified cells were injected intramuscularly. One week post injection, local confined plasmid expression and transient distributions of the plasmids in other organs were detected by quantitative PCR. Quantitative micro-CT analyses showed improvements of vascularization in the ischemic zone (No. of collateral vessels via micro CT: 6.8±2.3 vs. 10.1±2.6; *p*<0.05). Moreover, improved collateral proliferation (BrdU incorporation: 0.48±0.05 vs. 0.57±0.05; *p*<0.05) and increase in blood perfusion (microspheres ratio: gastrocnemius: 0.41±0.10 vs. 0.50±0.11; *p*<0.05; soleus ratio: soleus: 0.42±0.08 vs. 0.60±0.08; *p*<0.01) in the lower hindlimb were also observed.

**Conclusions:**

These results demonstrate the feasibility and effectiveness of double transplantation of gene nucleofected primary fibroblasts in producing growth factors and promoting the formation of collateral circulation in ischemic hindlimb, suggesting that isolation and preparation of gene nucleofected cells from individual accepting gene therapy may be an alternative strategy for treating limb ischemia related diseases.

## Introduction

Lower extremity ischemia causes many clinic disorders. Patients suffer from a slight muscle pain or walking problems to lower leg ulceration and gangrene or even amputation. Although surgical procedures can help some patients with arterial occlusions, new treatment approaches are still needed for the patients who are not suitable for surgery [1]. Therapeutic neovascularization based on proangiogenic growth factors has been suggested as a possible clinical approach [2], [3], [4], [5]. In this regard, the use of recombinant proangiogenic growth factors has been tested in both pre-clinic and clinical trials in previous studies. For example, Baffour R et al. proved that recombinant bFGF could enhance angiogenesis and growth of collaterals by *in vivo* administration in a rabbit hindlimb ischemic model [6]. In addition, VEGF was found to improve neovascularization in a hindlimb ischemia model [7]. However, although some of the results were encouraging from the past researches and randomized placebo-controlled double-blind clinical trials with recombinant proteins, there are still some sub-optimal results which are needed to be addressed. In particular, Lederman et al. reported that an improvement occurred in peak walking time 90 days after treatment in TRAFFIC trial with bFGF. However, this therapeutic effect was not observed at other time points [8]. In a randomized VEGF clinical trial (RAVE trial) with AdVEGF-121, there was no clinical therapeutic effects observed [9]. One of the possible explanations for failure of growth factor delivery in those studies could be owing to the direct administration of single growth factor, which could not induce enough therapeutic effects.

The combined administration of both bFGF and VEGF has been tested recently and found to have synergistic effects. In 1995, Asahara et al. proved for the first time that such synergistic effects of bFGF and VEGF enhanced collateral growth in a rabbit hindlimb ischemia model when they were administrated with a ratio of around 1∶50 (bFGF∶VEGF) [10]. However, the usual limitations of protein therapies are the low half life of the recombinant proteins (VEGF: 3 to 6 minutes *in vivo* and bFGF 1.5 minutes to 3 minutes *in vivo* [11], [12], [13]) and the possible side effects associated with high doses of exogenous proteins (apart from the possible tumorigenic effects because of inducement of angiogenesis, high dose VEGF could cause edema due to an increased microvascular permeability[14], [15], high dose bFGF could cause hypotension related to a dose-dependent vasodilating effect [16]).

Currently, gene therapy has emerged as a rational approach to constantly produce and release proangiogenic molecules in the ischemic area [17], [18], [19]. In this regard, Lee et al. demonstrated that injection of both bFGF and VEGF plasmids together intramuscularly improved the therapeutic effects in ischemic damage. However, the short biological half life of the both factors owing to the immune clean effects was found to lead to failure of long-term therapeutic efficacy. To improve the efficiency of gene transfection, many previous studies used viral vector to enhance transfection efficiency by direct delivery of gene construct or via injection of cells transfect with viral vectors. Kondoh et al. reported that virus-based *ex vivo* gene transfer method [20] increased transfection efficiency; in addition, high efficiency of adenovirus-based *ex vivo* fibroblast gene transfer was also established for bFGF and VEGF genes. However, owing to the biohazard associated with the use of viral vectors, viral gene therapy is not yet widely accepted for clinical use.

In the present study, we developed a new strategy using *ex vivo* gene transfer of autologous cells via non-viral nucleofection technique to maximize the bFGF and VEGF expression in *in vitro* and *in vivo* experiments. This strategy was proven to have the highest transfection efficiency when compared to other classic non-viral methods, including the original nucleofection method. In the animal model of hindlimb ischemia, the delivered primary fibroblasts remarkably increased the formation of collateral vessels and improved blood supply to ischemic tissue area, as measured by micro-CT 3D reconstructions of microvascular networks and quantification of arteriogenesis and angiogenesis.

## Materials and Methods

### Cell isolation and characterization

Fibroblasts were isolated from skin obtained from the back of inbred rats. Briefly, the samples were cut into 4 cm×1 mm strips and then incubated with Dispase-2 (Roche, Penzberg, Germany). Subsequently, the epidermal layer was carefully removed and the dermis was minced and then incubated for 3 hours under magnetic rotation with 0.1% collagenase (Roche, Penzberg, Germany). After that, the mixture was filtered through a 100-µm Cell Strainer (BD biosciences, Hamburg, Germany) to obtain the primary cells. For characterization, cytospinned cells and normal cells seeded for 48 hours were fixed in ice-cooled ethanol for 30 minutes. Subsequently, the cells were stained with anti fibroblast antibody (Prolyl 4-hydroxylase subunit beta: P4H beta, Acris, Heidelberg, Germany) and tetramethyl rhodamine isothiocyanate conjugated phalloidin (Sigma-Aldrich, MO, USA) according to the manufacturer's instructions. Then, the cells were mounted in Prolong-containing DAPI (Invitrogen, Oregon, USA). Afterwards, cell morphology was analyzed by phase-contrast/fluorescence microscopy (Nikon, eclipse te2000-s).

### High-efficient *ex vivo* nucleofection

Nucleofection was performed under different conditions, and 1×10^6^ primary rat skin fibroblasts (passage 2, 80–90% confluent) were trypsinized and harvested by centrifugation at 100×g for 10 min. The original nucleofection protocol was conducted following the suggested instructions (Basic Primary Mammalian Fibroblast Nucleofector® Kit, Lonza, Cologne, Germany). The supernatant was removed and the cell pellets were resuspended in 100 µl of nucleofection solution or 100 µl of Dulbecco's Modified Eagle Medium(DMEM) supplemented with 10% Fetal Calf Serum (FCS) for the purpose of comparing different nucleofection solutions. Afterwards, 4 µg of pmaxGFP® plasmids (Lonza) were mixed with the cells and subsequently transferred into nucleofection cuvettes. We also tested different transfection cuvettes and found that Eppendorf electroporation cuvettes (4-mm gap, Eppendorf, Hamburg, Germany) were as efficient as Amaxa cuvettes (2-mm gap, Amaxa, Cologne, Germany). Subsequently, the cells were nucleofected by using the U30 program from the nucleofection device and 500 µl of pre-warmed culture medium was immediately added to the cells. The cells were trypsinized and analyzed with a CASY® system (Innovatis AG, Reutlingen, Germany) for cell number and cell viability 48 hours after transfection. Transfection efficiency and apoptosis were quantified by fluorescent activated cell sorting (FACS; Cytomation MoFlo® Flow Cytometer, Dako, Denmark). DMEM+10% FCS, Eppendorf cuvettes (4-mm gap, Eppendorf, Hamburg, Germany) and U30 program were chosen for the *in vitro* and *in vivo* study. Plasmids encoding for VEGF165 and bFGF were constructed based on pmaxGFP® backbone (Lonza). The modified VEGF165 nucleofection protocol was as following: 1) Fibroblasts (3×10^6^, passage 2) were mixed with 12 µg of VEGF165 plasmid and 100 µl of nucleofection solution (DMEM+10% FCS); 2) the mixture was then transferred into Eppendorf electroporation cuvettes (4-mm gap) and nucleofected with single electropulse program U30. After that, cells were transferred into a T25 culture flask and 4 ml DMEM+10% FCS were added; 3) the above two steps were repeated for 5 times with a total of 15×10^6^ cells. 4) After 24 hours, VEGF165 nucleofected cells were collected and counted, only 5×10^6^ cells were obtained for *in vivo* administration. The bFGF nucleofection protocol was conducted at the same time as VEGF165 nucleofection with another 15×10^6^ cells to obtain 5×10^6^ bFGF nucleofected cells. Cells (5×10^6^ with bFGF and 5×10^6^ with VEGF165) were then mixed before *in vivo* cell administration.

### Growth-factor production

Transfected cells (1.4×10^5^) were seeded in 12-well plates and cultured in 1.5 ml of DMEM + 10% FCS. Subsequently, medium was changed and collected daily. Concentrations of VEGF165 and bFGF were quantified by Bio-Plex Suspension Array System, following manufacturer's instructions (Bio-Rad, Hamburg, Germany).

### Hindlimb ischemia model

Experiments were performed on male Lewis inbred rats (weight 200 grams, Charles River Laboratories, Sulzfeld, Germany). Femoral artery ligation was performed as described previously [21]. All *in vivo* procedures were approved by animal committee of Luebeck University (No.1/1m/09).

### Cell administration

Animals were randomly divided into 2 groups of treated and control animals. Directly after ligation of the right femoral artery, the cells (1×10^7^) transfected with VEGF165 or bFGF (5×10^6^ each) were intramuscularly injected into the gracilis muscle and adductor muscles in the middle part of the thigh because it has been demonstrated that this is the site of major collateral growth in the rat model employed but also in other animal models [21], [22]. This is also the site of major collateral growth in humans when the superficial femoral artery is occluded and was consequently chosen as main injection site in the TAMARIS and WALK trials [23]. The same number of cells without transfection was injected into the same site in control group.

### Quantitative micro-CT system

High-resolution desktop X-ray micro-CT system (SkyScan1072, SkyScan, Belgium) was used to visualize and quantify the vascular networks. Seven days after femoral artery ligation, micro-CT angiographies were performed as described before with modifications [21], After perfusion with the contrast medium, the ischemic zone of limbs was reconstructed from Z-axis cross section slices. After visualization and reconstruction of the vascular networks in the ischemic zone, collateral vessels were quantified and compared between the control and treated animals as described earlier [21]. Typical corkscrew-like collateral vessels were counted in 3D view. Compared to X-ray view, collaterals were easier to identify. After 3D reconstructions, the bone structures were subtracted and the vessel volume was quantified. Voxel number information from reconstruction slices of the ischemia zone (600 cross-sections below the proximal end of the ligation point) was collected for quantification of blood volume (see Materials and Methods S1 for detailed protocol).

### Plasmid pharmacokinetics

The distribution of the plasmid was assessed by real-time PCR detecting the pmaxGFP® plasmid backbone sequences. At day 3, 7, 14, and 28 after injections, animals were sacrificed and organs were carefully collected and frozen for plasmid detection. Afterwards, the DNA was isolated with NucleoSpin® Tissue kit (MACHEREY-NAGEL, Dueren, Germany) and detected with specific primer for the plasmid backbone (see Materials and Methods S1 for detailed protocol).

### Local gene expression

Seven days post injection, animals were sacrificed and samples were obtained from the muscles of the treated and control animals. Total RNA was then isolated with NucleoSpin® RNA II kit (MACHEREY-NAGEL, Dueren, Germany) according to the manufacturer's instructions. VEGF165 and bFGF mRNA expression was detected with specific primer for both the genes (see Materials and Methods S1 for detailed protocol).

### Collateral proliferation and blood perfusion ratio

Animals were sacrificed, the middle zone of the collaterals on day 7 including the surrounding tissues were taken, and proliferation assays were performed as described previously [21], [24]. Proliferation of the collateral artery was detected by 5-Bromo-2′-Deoxyuridine (BrdU) labeling and detection Kit 2 (Roche Diagnostics, Penzberg, Germany). The proliferative index was calculated as the number of BrdU-positive nuclei to the total number of nuclei inside the vessel wall. Blood flow in the lower hindlimb was detected on day 7 after ligation by FluoSpheres polystyrene microspheres (15 µm, red-fluorescent, Invitrogen, USA). The microspheres were injected via catheters through the carotid artery of rats running on a treadmill. After injection, rats were sacrificed and gastrocnemius and soleus from both hindlimbs were taken. Blood flow was expressed as ratios of occluded and non-occluded hindlimb perfusion (microspheres numbers) of the soleus and gastrocnemius (see Materials and Methods S1 for detailed protocol).

### Statistical analysis

All data were evaluated by at least 3 independent experiments. The data were shown as Mean ± SEM. Statistical comparisons between 2 groups were performed with two-tail Student's *t*-test. One-way ANOVA followed by post-hoc analyses was used for comparison of differences within multiple groups. The differences between the groups were considered significant when *p*<0.05.

## Results

### Isolation and characterization of primary dermal fibroblasts

**Figure 1:**
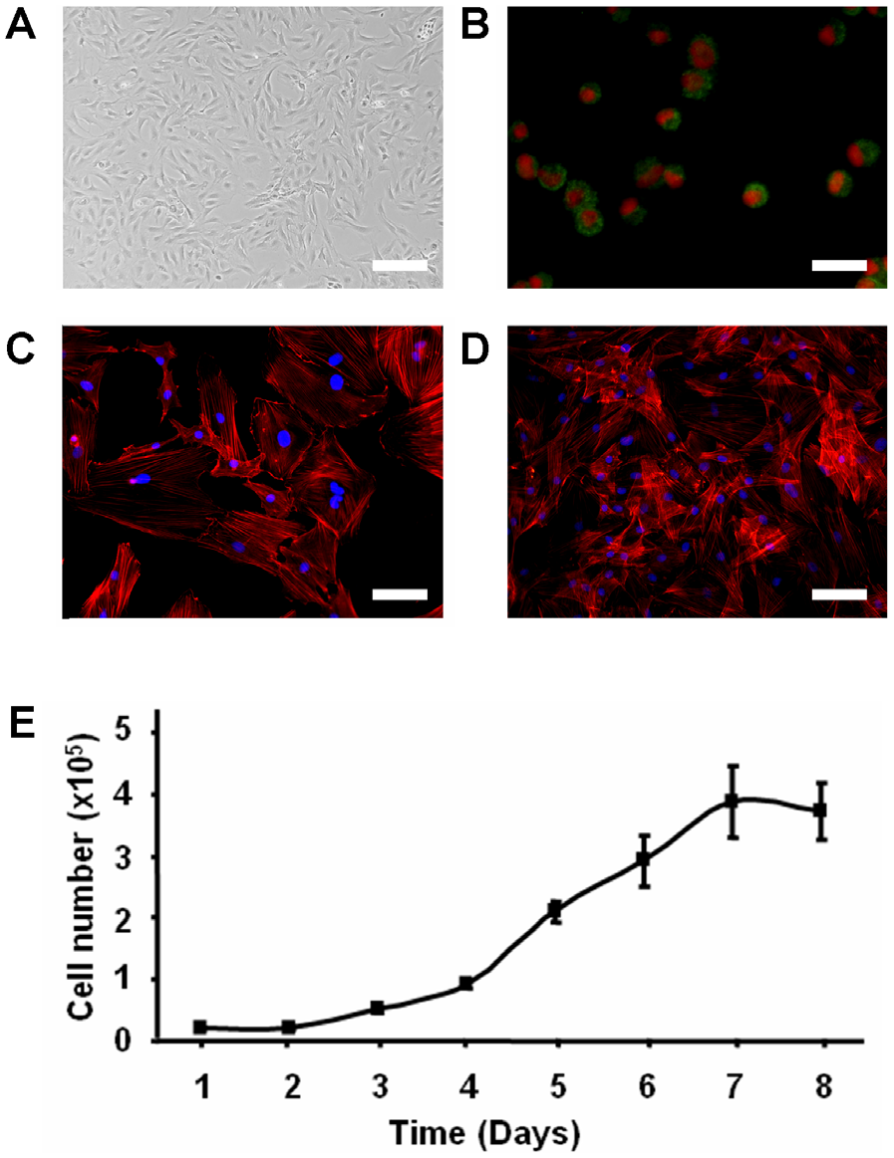
Preparation and characterization of rat primary fibroblasts. (A) Morphology of rat dermal primary fibroblasts (passage 2) was shown under phase contract microscope. (B) Characterization of isolated fibroblast with anit-fibroblast antibody (Prolyl 4-hydroxylase subunit beta: P4H beta), green: P4Hbeta; red: PI nucleus staining. (C) Morphology of fibroblasts under subconfluent and (D) confluent conditions was analyzed by phalloidin (red) and DAPI (blue) staining. (E) Primary rat dermal fibroblasts (passage 2) growth curve. Scale bar represents 200 µm in A, 100 µm in B and D, 50 µm in C.

Isolated rat dermal fibroblasts were spindle-shaped and adhered rapidly to the culture flasks (Figure 1A). The characteristics of these primary fibroblasts were detected by cytospin for the expression of prolyl 4-hydroxylase subunit beta (P4H beta). A fluorescent microscopic imaging for the expression of P4H beta is presented (Figure 1B). In addition, the actin cytoskeleton morphology of the fixed and permeabilized cells was also examined by FITC-label phalloidin under sub-confluent and confluent conditions (Figure 1 C and D), which indicated the nature of smooth muscle cells. The cell growth curve was obtained with normal culture medium (DMEM+10%FCS), showing an active cell replication with a doubling time of 24–48 hours. Cell growth was linear during the first 7 days and then reached a plateau phase on day 8 (Figure 1E).

### Optimization of nucleofection with improved reagents

We first examined transfection efficiency with six nucleofector programs (including A24, T16, U12, U23, U30, and V13). The cells were transfected with commercial protocol provided by the manufacturer. The highest transfection efficiency (around 60%) and cell viability (>95%) were obtained with program U30. Therefore, program U30 was chosen for further *in vitro* and *in vivo* experiments.

**Figure 2:**
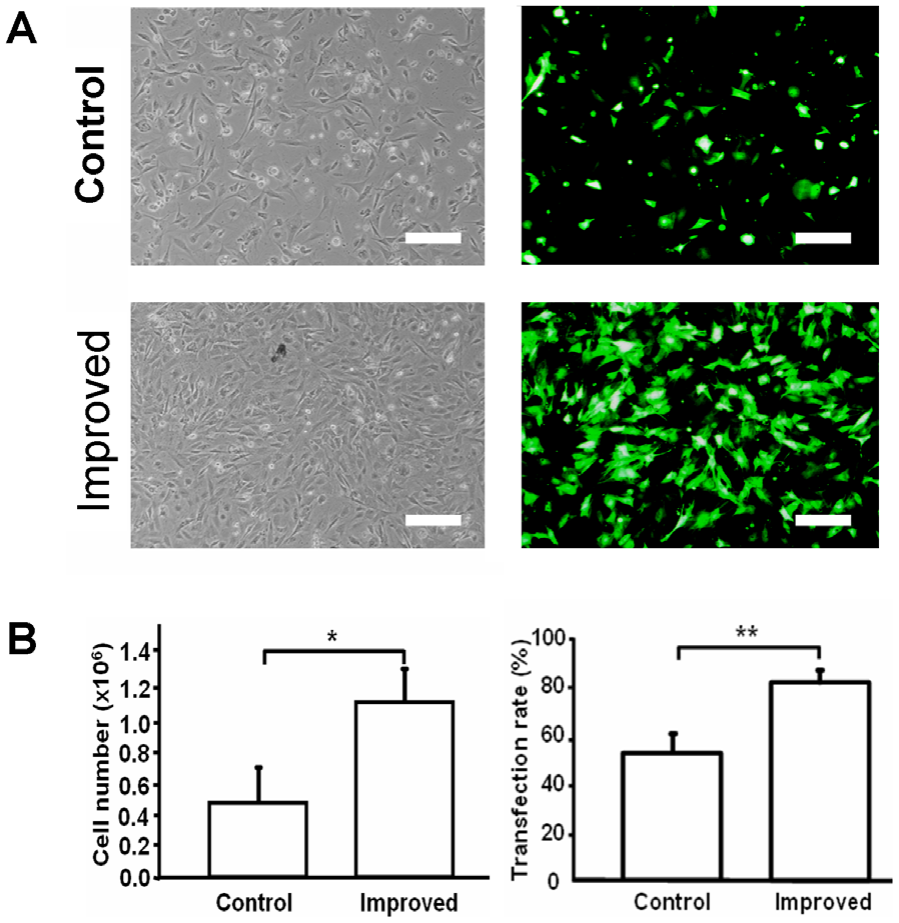
Optimization of nucleofection in primary cultures of fibroblasts. (**A**) Improved method (improved) and original method (control) were compared under fluorescence microscope to show optimization of nucleofection. Representative pictures of each method are shown in this panel. Scale bar represents 100 µm. (**B**) Cell number and transfection rate were significantly enhanced by the improved method (experiment was repeated for more than 5 times; * *p*<0.05; ** *p*<0.01).

To achieve the maximal therapeutic effects, nucleofection reagents were modified to improve gene transfection efficiency in primary cell cultures of fibroblasts (details described in Materials and Methods). Two days after nucleofection, the total number of cells significantly decreased in the control group, in which commercial kit reagents were used (Figure 2A, upper panels). When transfected reagents were modified, transfection efficiency, as shown by more GFP-positive cells, was enhanced (Figure 2A, lower panels). With such improved reagents, 2 days after nucleofection, more transfected cells were obtained in contrast to the control standard protocol (Figure 2B, left panel: 0.42±0.35 (Improved) vs. 1.07±0.29 (Control); *p<0.05). By FACS, we found that the use of modified solution did not affect cell growth, but induced higher transfection rate (Figure 2B, right panel: 80. 5±5.0 (Improved) vs. 51.5±7.9 (Control); **p<0.01).

### Dermal fibroblast nucleofection with VEGF and bFGF plasmid

**Figure 3:**
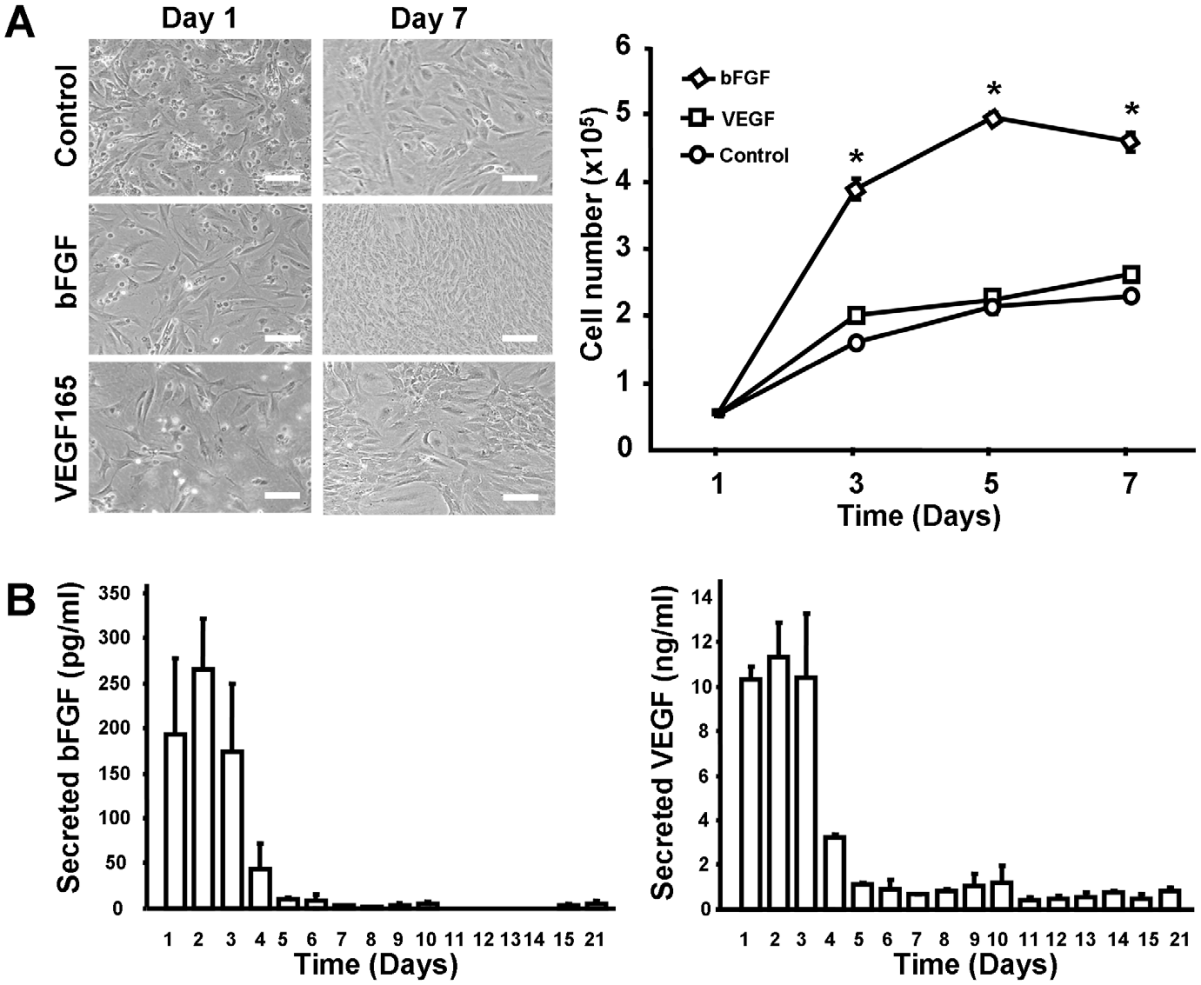
Cell growth and gene expression in primary cultures of fibroblasts transfected with bFGF and VEGF plasmids. After Nucleofection, 1.4×10^5^ cells were seeded in a 12 wells plate, 1.5 ml of the culture medium was changed every day. (**A**) 7 days after Nucleofection, more cells were observed in transfected groups. Quantification showed that significant differences were found between bFGF nucleofected group and control group (* *P*<0.05). (**B**) Recombinant growth factors were detected for up to 21 days in the culture media. Scale bar represents 100 µm.

After optimization with pmaxGFP vector, VEGF165 and bFGF sequences were inserted into the pmaxGFP® backbone as coding sequences. Seven days later, significantly more cells were observed in the group transfected with plasmids expressing bFGF when compared with the control (Figure 3A). Next, the growth-factor release was evaluated daily for 21 days (Figure 3B). Long-term expressions of both VEGF165 and bFGF proteins were found after nucleofection, and the dynamics of their release were similar. Both growth factors reached the expression or release peak at the first week. However, the amount of secreted growth factors was different between both the groups, with about 50-fold higher release of VEGF165 than bFGF.

### *In vivo* gene-delivery efficiency by nucleofected primary dermal fibroblasts

**Figure 4:**
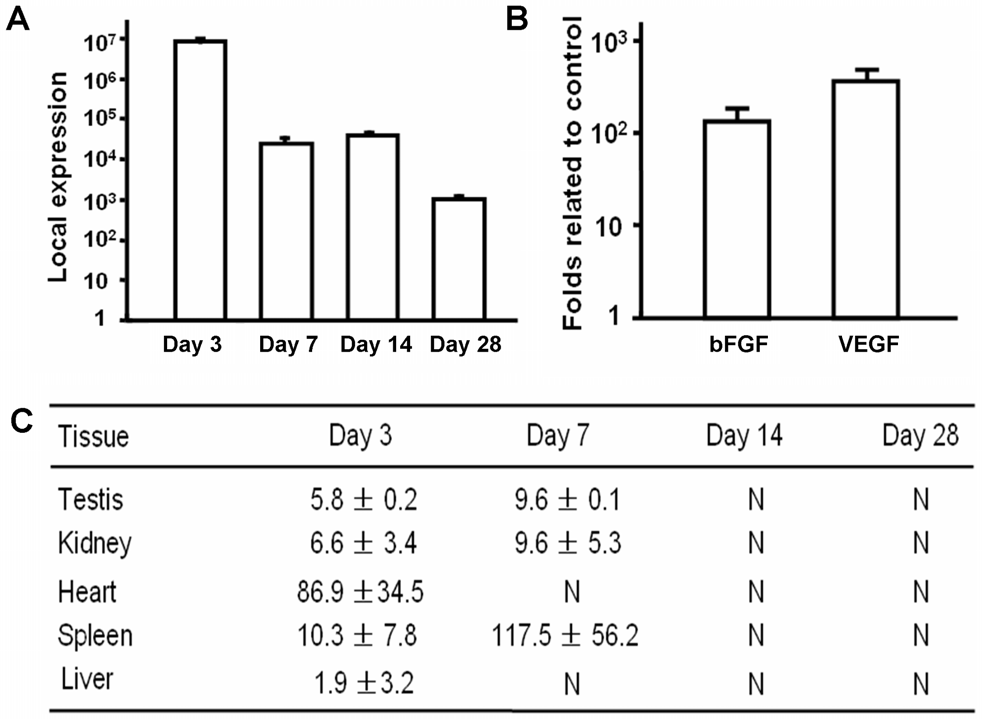
Gene delivery efficiency and expression *in vivo* by modified primary dermal fibroblasts. (**A**) High levels of plasmids were detected in the injected area for more than 28 days. (**B**) 7 days after cell injection, gene expressions of bFGF and VEGF165 from injected area were measured. Results showed that the expressions of both bFGF and VEGF165 were 100 times increased related to the control group. (**C**) After cell administration, organs or tissues were collected at 4 different time points (experimental group: n = 3; control group: n = 3) and the sequence of the plasmid backbone was used for detection of its distribution. Results are shown as the average value obtained from 100 ng DNA of each organ or tissue. N represents that no plasmids were detected.

After administration of genetically modified fibroblasts, distribution of the plasmids in the peripheral tissues or organs was detected by real-time PCR. No plasmid could be detected in any organs of the control rats injected with nonmodified cells. The plasmid was detected for more than 28 days at the site of injection (Figure 4A). The peak of expression at the injection site was on day 3, and then remained at a fairly high level. From day 3 to day 7, the plasmid concentration dropped rapidly. After day 7, the local concentration was maintained at the level of 10^5^ copies/100 ng DNA, and then deceased to the order of 10^3^ copies/100 ng DNA on day 28. Expression of bFGF and VEGF165 was also detected by PCR at the site of injection. On day 7 post injection, the expression of both angiogenic growth factors in the muscles was more than 100-folds higher than that in the control group (Figure 4B). In all peripheral tissues and organs from the experimental rats, no plasmid was detected after day 14 (Figure 4C).

### Administration of nucleofected fibroblasts enhanced angiogenesis and arteriogenesis in the ischemic zone

**Figure 5:**
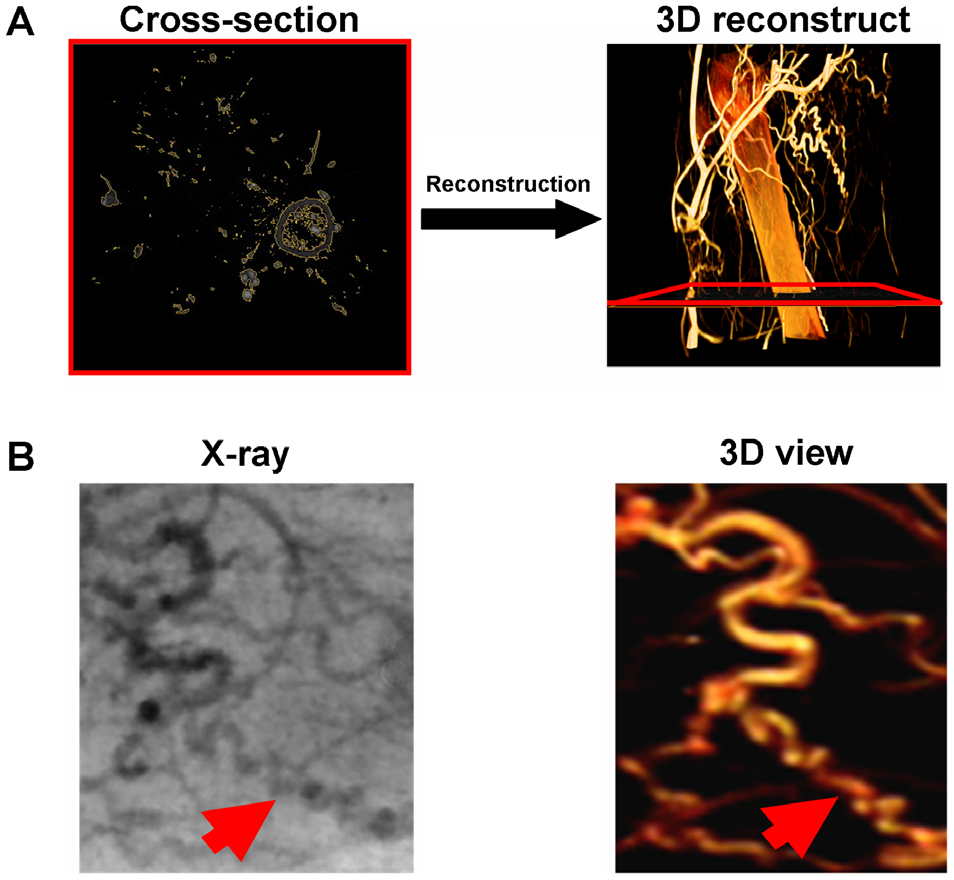
Comparison of micro-CT 3 dimensional collateral detection with planar method. (**A**) Ischemic hindlimb was reconstructed through 1024 Z-axis slices (left panel). (**B**) Vessels in 3 dimensional (3D) reconstruction views were analyzed. Red arrows show the same corkscrew collateral vessel in both X-ray review and 3D reconstruction view.

**Figure 6:**
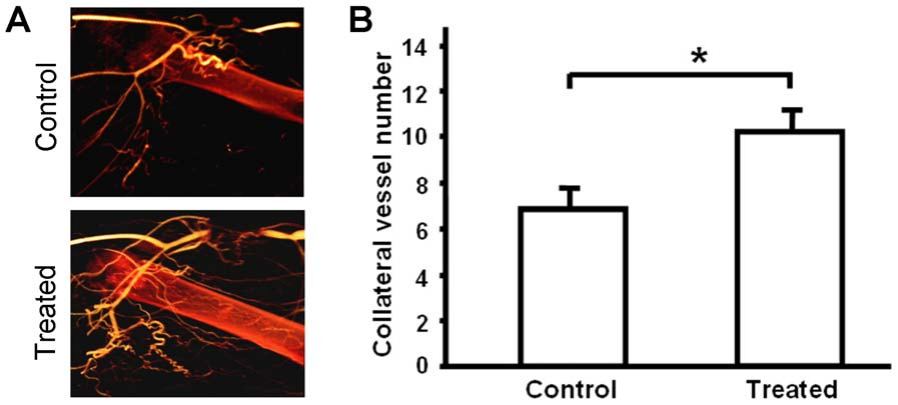
Increased 3D collateral vessels in ischemic hindlimbs treated with nucleofected fibroblasts. (**A**) One week after cell administration, micro-CT angiography was performed and the ischemic zone from both treated and control samples were reconstructed. (**B**) Summarized data showed that injection of transfected cells increased the number of visible collateral vessels (treated group: n = 7; control group: n = 6 *p<0.05).

**Figure 7:**
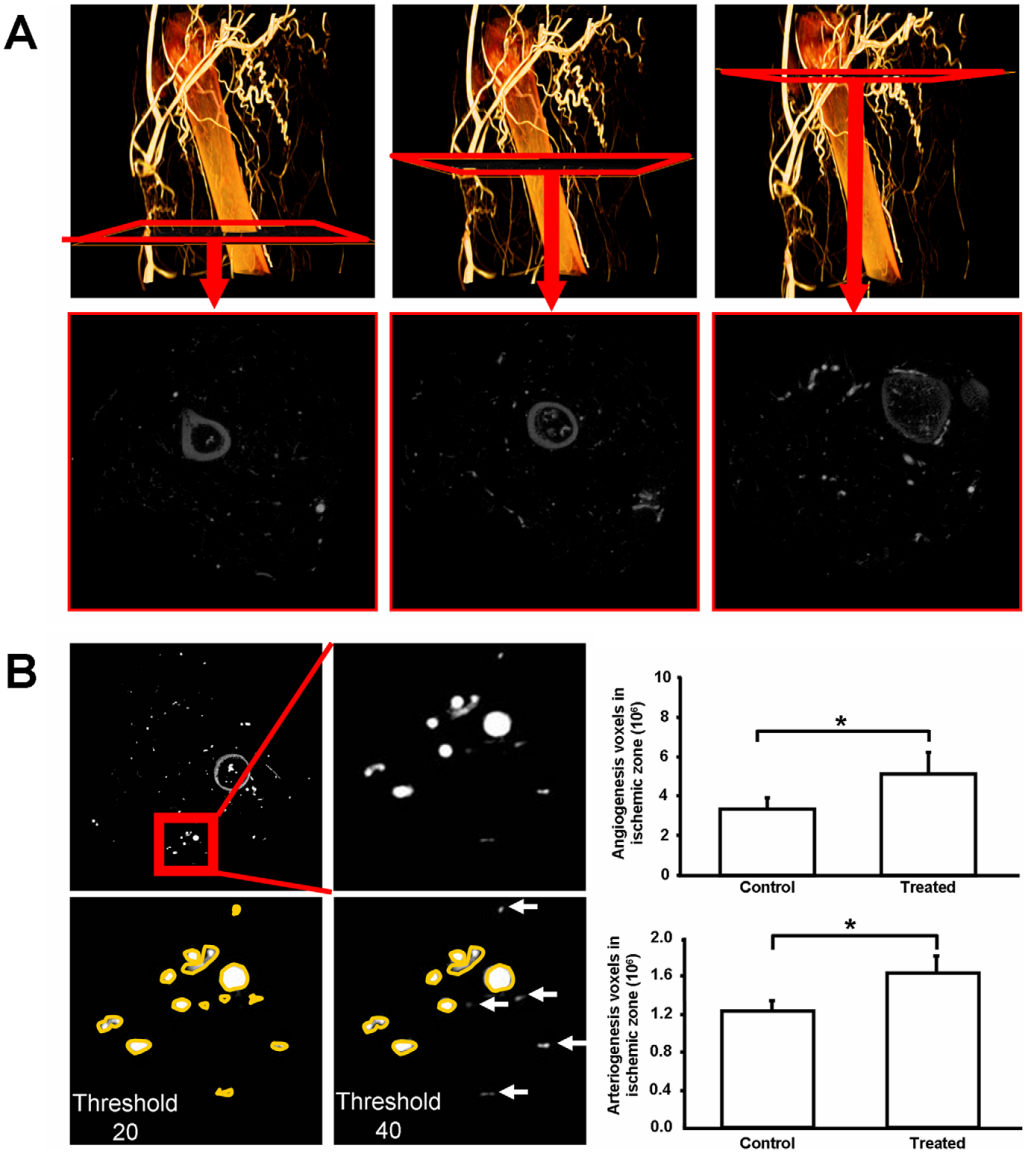
Increased vascular volume in ischemic hindlimbs treated with nucleofected fibroblasts. (**A**) The ischemic zone was defined as 600 slices below the proximal ligation point (slices from the left panel to right panel), including the distal ligation point in the middle panel. (**B**) Quantification was done with 2 different thresholds: T20 and T40. With threshold 20, all vessels (angiogenesis and arteriogenesis related) were counted. Threshold 40 was used to quantify only vessels with higher caliber (arteriogenesis related). (**C and D**) Vessels related to angiogenesis and arteriogenesis were significantly enhanced after nucleofected fibroblasts injection (treated group: n = 7; control group: n = 6; Angiogenesis: * *p*<0.05, Arteriogenesis: *p<0.05).

After cell administration, the ischemic zone was reconstructed (Figure 5A) and the collateral was counted in 3D reconstruction views (Figure 5B). A significant increase in the number of vessels was triggered by the injection of nucleofected cells (Figure 6A and B; *p<0.05). Quantification was conducted after reconstruction of the ischemic zone (Figure 7A). Both angiogenesis (Figure 7B, right upper panel; 3.61±0.56 *vs.* 5.13±1.08 *p<0.05) and arteriogenesis (Figure 7B, right lower panel; 1.33±0.09 *vs.* 1.63±0.06, *p<0.05) increased, indicating an improvement in the medium-size vessel and related small and thin vascular networks.

### Increased collateral growth and blood flow in ischemic hindlimbs treated with nucleofected fibroblasts

**Figure 8:**
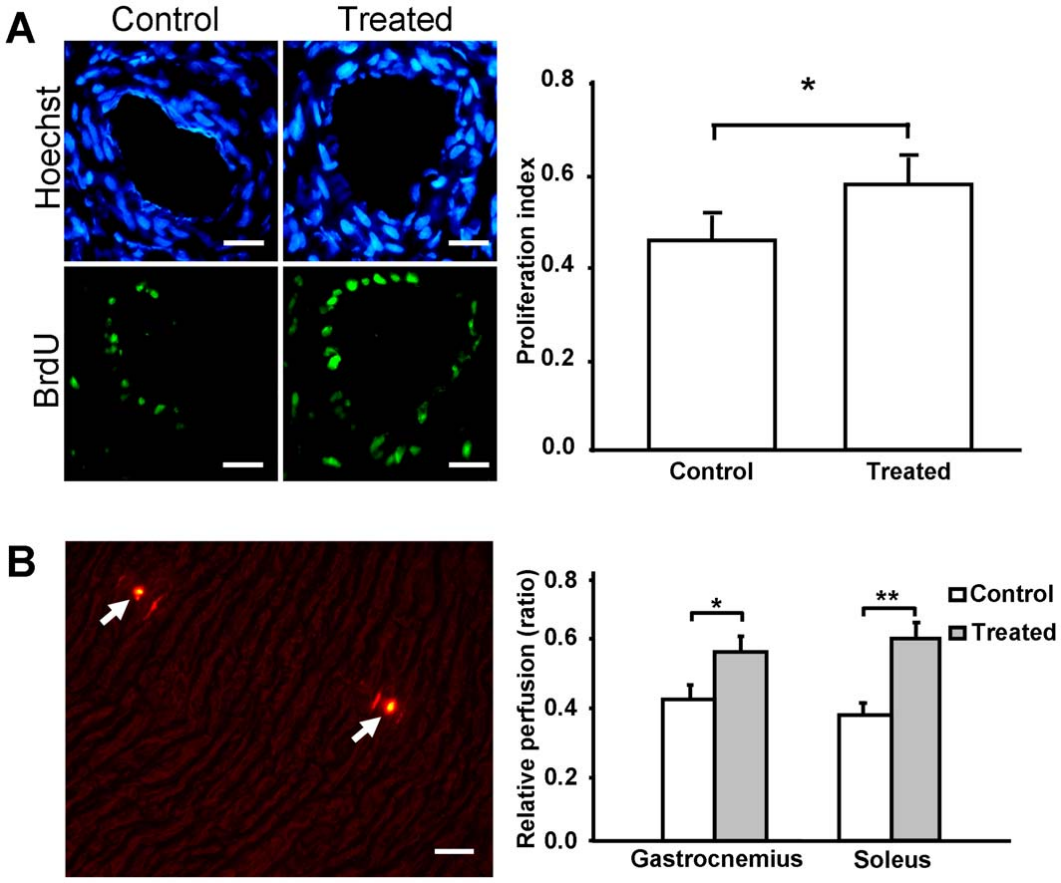
Increased collateral growth and blood flow in ischemic hindlimbs treated with nucleofected fibroblasts. (**A**) One week after cell administration, collateral growth was detected and quantified by BrdU and Hoechst staining. The proliferation index is expressed as BrdU (green) positive nuclei *vs.* total (Hoechst/blue). Quantification showed that proliferation index was significantly enhanced by the injection of transfected cells (Experimental group: n = 8; control group: n = 7,* *p*<0.05). Scale bar represents 50 µm. (**B**) Blood flow was analyzed by FluoSpheres® blood flow detection method. Cryosections were analyzed by fluorescent microscopy and the numbers of the microspheres per muscle sample were counted (left panel). The white arrows show the fluorescent microspheres trapped in the small capillaries. Blood flow ratio was calculated as numbers of microspheres trapped in the ligated muscle *vs.* the number trapped in the same part of the non-ligated leg in the same animal. Results are shown as gastrocnemius perfusion ratio between control and experimental group; soleus perfusion ratio between control group and experimental group (right panel). In both muscles, the blood flow was significantly improved (Experimental group: n = 8; control group: n = 7 soleus: **p<0.01 gastrocnemius *p<0.05). Scale bar represents 50 µm.

To further examine the effects of nucleofected cells on collateral growth, collateral proliferation index was analyzed after local administration of nucleofected cells. The results showed an enhanced collateral proliferation in the same model (Figure 8A, BrdU incorporation: 0.48±0.05 vs. 0.57±0.05; *p<0.05). Re-establishment of a collateral circulation in ischemic lower hindlimbs was detected by fluorescent microspheres. These microspheres were trapped inside the lumen of the small arteries and the number of the microspheres inside each muscle is related to the blood flow (Figure 8B, left panel). Microspheres from both soleus and gastrocnemius (ligated and nonligated hindlimbs) were counted under fluorescent microscope to obtain the perfusion ratio from each animal. Blood flow from soleus and gastrocnemius significantly increased after administration of nucleofected cells (Figure 8B, right; microspheres ratio: gastrocnemius: 0.41±0.10 vs. 0.50±0.11; *p<0.05; soleus ratio: soleus: 0.42±0.08 vs. 0.60±0.08; **p<0.01).

## Discussion

Although several technologies of gene therapy have been extensively applied in animal models of ischemia, their use in clinical settings is still limited [25]. Among the main reasons behind the poor clinical translation of animal studies into humans is the lack of both a highly efficient gene delivery system and relatively easy procedures for the clinical translation. Here, we described a modified nucleofection technology based on alternative reagents which owns a high transfection efficiency (>80%) and also a relatively easy operation procedures.

Nucleofection technology is a newly developed nonviral transfection method based on electroporation technology. It could be useful for applications in gene therapy with autologous cells [26], [27], [28]. Different approaches have been used for nucleofection based gene transfer into primary culture cells [29], [30]. Several previous studies have demonstrated the possibility of using this technology to promote neovascularization in ischemic tissues. Aluigi et al. have reported that nucleofection of human mesenchymal stem cells *in vitro* could produce gene expression for more than 20 days [26]. Although stem/progenitor cells were considered to be better candidates for *ex vivo* transfection, uncontrolled differentiation that could lead to a serious side effect was always a concern. Thus, in our study, the physiological stable autologous fibroblasts were used to deliver targeted genes into the ischemic tissue to demonstrate the efficiency of gene therapy. Compared with a recent study by Mueller et al., who demonstrated that a high-efficiency *ex vivo* rat dermal fibroblast transfection with VEGF nucleofection technology could induce local neovascularization effects in a rat skin flap model [31], our findings at least overcame two major drawbacks, namely the relative high cost (Our method cost only 10 folder less than the original method) and the low transfection efficiency (550 pg/ml peak expression of VEGF in the previous study[31] and 10 ng/ml peak expression of VEGF in our present study). Thus, improved reagents for nucleofection in the present study will largely reduce the cost for high efficiency of nucleofection. This modified nucleofection may direct toward the development of similar protocols for human autologous cell gene therapy. However, it should be noted that much work may be needed to translate the findings from the present study to a clinical setting. Most importantly, there is a need to establish efficient protocols for preparation of primary cell cultures from human tissues and to establish the nucleofection efficiency of related genes into such cell cultures. In addition, the applicability for different ischemic diseases in human needs more careful evaluation because the ischemic changes or stages observed in the animal model we used in the present study may be not necessarily seen under human conditions. Certainly there are a number of promising candidates and promising ways of therapeutic application [32], [33], [34], [35]. In the light of previously published studies, the complex mechanism of collateral growth and last not least the results of the present study we think a gene therapy with combination of different growth factors may be likely to have a sustained effect in the treatment of lower extremity ischemia. However, as pointed out above, it is still a very long way to clinical application.

The delivery of our cells directly into the middle part of the thigh is also another important aspect which may favor the therapeutic effects while lower the possible systematic side effects. Our results also confirmed a possible localized expression pattern. It was found that the plasmid can be detected for more than 28 days in the injected area, but it was undetectable in other organs after 14 days. The peak of expression of the plasmid *in vivo* was determined to occur on day 3 (Figure 4A), which is consistent with the *in vitro* data (Figure 3B). Although most of the previous studies showed that less than 10% of the plasmid can reach the general circulation after direct plasmid intramuscular injection [36], [37], it was reported that plasmid can be detected by PCR up to 1 year in mice [38]. Here, we showed that the use of *ex vivo* transfection has substantially decreased this time. When compared with the local plasmid concentration, the plasmids in the other organs were fairly low. On day 7, plasmid accumulation in the spleen could be explained by “cleaning effects” of the circulating plasmid by the immune system. All these findings indicate that autologous cell gene delivery can make sustained gene expression in the injected area locally.

After injections of gene-modified fibroblasts into an ischemia model, we also analyzed the neovascularization effects using a 3D micro-CT system. This technology was used here because it presents several advantages when compared to the planar area measurement methods. In our previous studies, angiographies had to be taken from different angles to obtain sufficient information about the collateral vessels [21], [24]. Even under the best circumstances, reliable collateral vessel counting is difficult to perform. In the present study, we were able to easily reconstruct the 3D structures of the ligated hindlimbs and quantify the collateral vessels in 3D views (Figure 5A and B). With the use of this method, we were also able to quantify the blood volume by voxel rendering in the reconstruction sections. Vessels related to arteriogenesis and angiogenesis were analyzed separately (Figure 7), and both were found to be improved after cell administration. This result was confirmed by measuring the blood flow in lower hindlimb (Figure 8). Using this technique, we analyzed the efficiency of this autologous cell therapy with gene delivery in the hindlimb ischemia model. Our results strongly suggest that autologous cell therapy with mixture of VEGF165 and bFGF nucleofected cells is very efficient to promote the formation and growth of collateral vessels and re-establish circulation in ischemic area of hindlimb.

In summary, we established a gene delivery method based on highly efficient modified nucleofection approach using double transplantation of nucleofected primary fibroblasts. This technology owns 2 major advantages over other local application: 1) No exogenous vectors are needed; 2) High transfection efficiency with a relatively low cost. This technology might have the potential to be used in the future clinically.

## Supporting Information

Materials and Methods S1 Detailed protocols of quantitative micro-CT system, plasmid pharmacokinetics, local gene expression and collateral proliferation index and perfusion index detection.
(DOC)
