# Supplementary material for: Enhanced Collateral Growth by Double Transplantation of Gene-Nucleofected Fibroblasts in Ischemic Hindlimb of Rats
Source: PLoS One. 2011 Apr 25;6(4):e19192. doi: 10.1371/journal.pone.0019192 (PMC3081850; doi:10.1371/journal.pone.0019192)
Supplement: Materials and Methods S1 — Detailed protocols of quantitative micro-CT system, plasmid pharmacokinetics, local gene expression and collateral proliferation index and perfusion index detection. (DOC) [file pone.0019192.s001.doc]

**Materials and Methods S1**

**Quantitative micro-CT system**

Twelve grams of gelatin type A from porcine skin (Sigma) and 60 g of barium sulfate (Merck) were dissolved in 100 ml of heated distilled water under continuous steering. Seven days after femoral artery ligation, the rats were anesthetized and anticoagulated with heparin (100 IU/L), and the aorta was annulated. Subsequently, the vasculature was flushed with 0.9% normal saline (37°C at 250 mm Hg pressure), and then with neutral buffer containing 10% formalin. After the clearance of blood from the no-ligation leg, the rats were put into warm water (37°C), and contrast medium was then injected with the pressure of 250 mm Hg until filling of the distal femoral stump was observed. Then, the animals were immediately placed on crushed ice, and contrast medium was allowed to harden under continuous pressure. Afterwards, the ligated hindlimbs were cut off carefully and fixed with 4% formalin for 12 hours before micro-CT detection. The micro-CT system contains an 80-kV microfocus tube with a focal spot size of 8 µm. Its imaging system consists of an X-ray source and a 2D X-ray detector creating projection images (cone beam reconstruction mode). The field of view is vertically adjustable by translation of the sample holder. The magnification is determined by the adjustable position of the sample holder between the detector and the X-ray source. The sample holder rotation step was set at a minimum of 0.45°, which corresponds to 400 views or projections. From these projection images, the virtual 3D Model of the sample is recreated. DataViewer (Version 1.3.2 SkyScan, Belgium) was used for the 3D Model visualization of the collateral vessels from all the projections, and the collateral vessels were visualized and counted in both original X-ray projections and 3D structures.

The reconstruction of the raw projections data was obtained by the NRecon reconstruction (v1.5.1, SkyScan). Here, the whole Z-axis was reconstructed. All the reconstructed sections were stored and used later for the counting of voxels per slice. Subsequently, the bone structure was subtracted from the image by feature extraction. Then, quantifications were done by the measurement of the voxel numbers in the ischemic zone. These numbers were obtained from the 600 cross-sections used to reconstruct the images, below the proximal end of the ligation area. We also quantified arteriogenesis and angiogenesis in the ischemic zone. The terms arteriogenesis and angiogenesis were used to discriminate different sizes and formation pattern of the vessels. Arteriogenesis quantification was focused on the quantification of the typical medium-sized collateral vessels, which might formed by changed shear force and local growth factors release after femoral artery occlusion[1,2]. Quantification of angiogenesis was focused on the thin-walled small-size vascular network. Both the quantifications were carried out by measurement of the voxel numbers in the ischemia zone from 600 cross-section reconstructed images below the proximal end of ligation (Figure 7A, left panel: first section crosses the proximal end of ligation; middle panel: section crosses the distant end of ligation; right panel: last section of the ischemic zone). We used two different thresholds, 20 and 40, from the Amira 4.0 software for such quantification. Threshold 20 was used to quantify both small-size and medium-size vessels (Figure 7B, T20), and threshold 40 was used to quantify only the medium-size vessels (Figure 7B, T40). Total voxel number of arteriogenesis was counted with threshold 40, and the total voxel number of angiogenesis was calculated by subtracting the value of threshold 40 from threshold 20.

**BrdU proliferation index**

After femoral artery ligation, the animals were supplied with osmotic minipumps (Alzet, Germany) filled with the thymidine analog, 5-bromo-2-deoxyuridine (BrdU; Sigma). Osmotic pump preparation was conducted according to the manufacturer’s instructions. Under sterile conditions, osmotic minipumps (model 2Ml1, 10 μl/ h, 7 days) were filled with 62 mg of BrdU dissolved in 3 ml of 0.5 M NaHCO_3_ buffer (pH 9.8), and implanted subcutaneously in the back of the animals. Seven days later, after the administration of the microspheres, the midzone part of the collateral artery including the surrounding tissue was removed, and samples were embedded in Tissue Tek (OCT compound, Sakura Finetek, Japan), and shock-frozen in liquid nitrogen. Then, cryosections (10 μm thick) were obtained and fixed in glycine and ethanol (3:7, pH 2.0) for 20 min at −20°C. After drying the sections for 30 min, the samples were washed in phosphate-buffered saline (PBS) and blocked for 30 min in 1% BSA (bovine serum albumin, Fluka). For detection of BrdU labeling, 5-Bromo-2’-Deoxyuridine Labeling and Detection Kit 2 (Roche Diagnostics, Germany) was used according to the manufacturer’s instructions. As second antibody, an FITC-conjugated goat anti-mouse IgG (Acris Antibodies GmbH, Germany) was used (1:500 in 1% BSA). Nuclear visualization was performed by Hoechst-3342 staining (Cambrex). Then, the samples were mounted in Mowiol (Calbiochem) and 1, 2-phenylenediamine (Merck), and analyzed by fluorescence microscopy. The proliferative index was calculated as the number of BrdU-positive nuclei (green fluorescence) to the total number of nuclei (blue fluorescence) inside the vessel wall. The numbers were obtained from five different random fields.

**Lower hindlimb blood perfusion detection with fluorescent microspheres**

Seven days after ligation, 15 µm of fluorescent-labeled microspheres (invitrogen, Germany) were infused by the left carotid artery. Here, a catheter (PE-50 tubing, Instech Laboratories, PA USA) was placed in the arch of the aorta, and then filled with heparinized saline (100 IU/ml). Afterwards, the catheter was fixed subcutaneously in the area of the neck. Three hours later, the animals were placed in a treadmill for running, and 0.45 ml of saline containing 450,000 microspheres was injected via the catheter. After running for 1 min (12 m/min at 15% grade), the animals were sacrificed. Then, the middle part of the collateral (for BrdU proliferation assay), gastrocnemius, soleus, and middle part of both the kidneys were immediately collected and embedded in Tissue-Tek (OCT compound, Sakura Finetek) and stored in liquid nitrogen. For microspheres detection, the cryosections (60 µm) were analyzed by fluorescence microscopy as described earlier [3,4,5]. Here, blood flow was expressed as the ratio between the number of microspheres in occluded and non-occluded hindlimbs in soleus and gastronemius. Samples with more than 15% differences in the content of microspheres between both the kidneys were excluded from the analysis.

**Plasmid distribution analyses**

After injection of 10^6^ control or transfected cells, animals were checked daily to observe any clinic side effects. At days 3, 7, 14 and 28, animals were sacrificed and tissues from different organs were carefully removed, shock freeze with liquid nitrogen and stored at -80 ℃ for further analysis. For each time point 3 animals treated with transfected cells were used. As negative control, 1 rat injected with non modified cells was analyzed. For DNA isolation, tissues were homogenized in liquid nitrogen and 25 mg of powder was used. DNA isolation was performed with NucleoSpin® Tissue RNA/DNA isolation kit (MACHEREY-NAGEL GmbH, Germany) following the manufacturer’s instructions. Final DNA concentration was detected by OD-260. Real-time PCR was carried out with a final volume of 25 μl , containing 100 ng DNA in 5 µl H_2_O, 12.5 µl iQTM SYBR® Green Supermix (Bio-Rad), 0.5 µl upstream primer, 0.5 µl downstream primer and 6.5 µl H_2_O. As positive control, 10^4^ pmax-VEGF plasmid molecules in 5.0 µl H_2_O were used. The primers for pmax plasmid backbone are as follows:

Forward: 5’-TGA AGC ATT TAT CAG GGT TCG-3’

Backward: 5’-CAA CAT GGC GGT CAT ATT GG-3’

**VEGF and bFGF expression**

RNA isolation was described in material and methods, 1 ng RNA was used to detect the expression of VEGF^165^ and bFGF. GAPDH was used as housekeeping gene. Next primers were used for RNA amplification:

bFGF: Forward: 5’-GGC TAT GAA GGA AGA TGG A-3’;

Reverse: 5’-CAG CTC TTA GCA GAC ATT G-3’

VEGF: Forward: 5’-GTG GAC ATC TTC CAG GAG TA-3’

Reverse: 5’-CTT TGG TCT GCA TTC ACA-3’.

MyiQ^TM^ Single-Color Real-Time PCR Detection System (Bio-Rad, Munich, Germany) was used for PCR detection. Real-time PCR was carried out with a final volume of 25 μl, with 1 ng cDNA, 12.5 µl iQ^TM^ SYBR® Green Supermix (Bio-Rad), 0.5 µl forward primer, 0.5 µl reverse primer and 6.5 µl H_2_O. PCR was performed with an initial denaturation at 95℃ for 3 min, cycling 40 times of 10 s denaturing at 95℃, 20 s annealing at 59℃ and 20s extension at 72℃. A melt-curve protocol immediately followed amplification with 95°C for 1 min and 59°C for 1 min, followed by 73 repeats of heating for 10 sec, starting at 59°C with 0.5°C increments. Ct values were automatically calculated with the data analysis module. Results were analyzed according to the delta-delta Ct method. Samples were collected from all experimental and control animals and results were expressed as gene expression ratio between experimental and control rats.

**References**

1. Pipp F, Boehm S, Cai WJ, Adili F, Ziegler B, et al. (2004) Elevated fluid shear stress enhances postocclusive collateral artery growth and gene expression in the pig hindlimb. Arterioscler Thromb Vasc Biol 24: 1664-1668.

2. Ito WD, Khmelevski E (2003) Tissue macrophages: "satellite cells" for growing collateral vessels? A hypothesis. Endothelium 10: 233-235.

3. De Visscher G, Haseldonckx M, Flameng W (2006) Fluorescent microsphere technique to measure cerebral blood flow in the rat. Nat Protoc 1: 2162-2170.

4. Jacobi J, Sydow K, von Degenfeld G, Zhang Y, Dayoub H, et al. (2005) Overexpression of dimethylarginine dimethylaminohydrolase reduces tissue asymmetric dimethylarginine levels and enhances angiogenesis. Circulation 111: 1431-1438.

5. Jacobi J, Tam BY, Wu G, Hoffman J, Cooke JP, et al. (2004) Adenoviral gene transfer with soluble vascular endothelial growth factor receptors impairs angiogenesis and perfusion in a murine model of hindlimb ischemia. Circulation 110: 2424-2429.
